# Promoting growth and production of sunchoke (*Helianthus tuberosus*) by co-inoculation with phosphate solubilizing bacteria and arbuscular mycorrhizal fungi under drought

**DOI:** 10.3389/fpls.2022.1022319

**Published:** 2022-10-31

**Authors:** Sabaiporn Nacoon, Wasan Seemakram, Jindarat Ekprasert, Sanun Jogloy, Thomas W. Kuyper, Wiyada Mongkolthanaruk, Nuntavun Riddech, Theerasak Somdee, Sophon Boonlue

**Affiliations:** ^1^ Department of Microbiology, Faculty of Science, Khon Kaen University, Khon Kaen, Thailand; ^2^ Department of Agronomy, Faculty of Agriculture, Khon Kaen University, Khon Kaen, Thailand; ^3^ Soil Biology Group, Wageningen University & Research, Wageningen, Netherlands

**Keywords:** jerusalem artichoke, inulin, rock phosphate, osmotic stress, plant growth promotion

## Abstract

Due to different functions of phosphate solubilizing bacteria (PSB) and arbuscular mycorrhizal fungi (AMF), their potential synergistic effects on enhancing plant growth and yield are worth investigating, especially under adverse conditions. This work focused on the isolation of PSB and characterization for their plant growth promoting properties under drought. The most efficient P solubilizing bacterium was isolated and identified as *Burkholderia vietnamiensis* strain KKUT8-1. Then, a factorial experiment on the performance of sunchoke (*Helianthus tuberosus*) was set up with four factors, viz., PSB (presence or absence of KKUT8-1), AMF (presence or absence of *Rhizophagus aggregatus*), rock phosphate (RP; added or not) and moisture (well-watered (WW) or drought (DS) conditions). Sunchoke performance was enhanced by the presence of AMF, whereas addition of PSB had a positive effect on SPAD values and inulin concentration. Drought reduced plant performance, while addition of RP reduced photosynthetic rate. There was little evidence for synergistic effects between PSB and AMF, except for SPAD values and inulin concentration. Plants that were co-inoculated with AMF and PSB had highest SPAD value, shoot diameter, leaf area, leaf number, chlorophyll concentration, plant biomass, tuber production, root growth and total soluble sugar concentration. Co-inoculated plants also had increased plant water status, reduced electrolyte leakage, and reduced malondialdehyde and proline concentration. Strain KKUT8-1 is the first strain of *B. vietnamiensis* capable of promoting growth and yield of sunchoke. Enhanced production of sunchoke by a combination of AMF and PSB was much better than the application of RP. Our finding offers an opportunity to develop combinations of biological inoculants for increasing the growth and production of sunchoke under drought in the future.

## 1 Introduction

Sunchoke (*Helianthus tuberosus* L.: Asteraceae), also known as Jerusalem artichoke, is a species related to sunflower (*H. annuus* L.) that is efficiently used for biorefinery purposes due to its high biomass and limited cultivation requirements for its growth. Both above and belowground parts of sunchoke can be used for various applications including food, medical applications, and ethanol production (Kiru and Nasenko, 2010). Sunchoke has been widely cultivated for its polysaccharides and a group of non-digestible carbohydrates, especially inulin, which accumulates in the tubers (Shoaib et al., 2016). However, due to water scarcity in many agricultural areas, sunchoke suffers from drought leading to a decrease in biomass and yield (Mahajan and Tuteja, 2005). Drought also disturbs several plant physiological processes such as disruption of membrane structure, inhibition of enzyme activities, and damage to ultra-structural cellular components (Hasanuzzaman et al., 2017). Plant growth parameters, plant production, and inulin concentration in sunchoke were afflicted when plants were grown under drought (Ruttanaprasert et al., 2016). Moreover, drought causes a reduction in phosphorus (P) acquisition, thus leading to low plant P concentration (Sardans and Peñuelas, 2004). As a result, several plant growth processes such as photosynthesis, carbohydrate metabolism, membrane formation, energy generation, nucleic acid synthesis, and respiration are reduced (Leidi and Rodríguez-Navarro, 2000). P deficiency under drought negatively affects both early and late stage of plant growth including root extension, seed development and crop maturity (Williamson et al., 2001). To overcome limited mobility of available P in dry soils, mineral phosphatic fertilizers are applied to fulfil plant P demand; however, mineral-fertilizer P usually becomes rapidly unavailable to plants, due to sorption to and precipitation with mineral soil (Wang et al., 2017). Due to those limitations of mineral fertilizers, recent research has focused on alternative sources of P fertilizer. Rock phosphate (RP) is an important natural source of P that is used as raw material for the production of mineral P fertilizers (Reddy et al., 2002). Although RP is a mineral fertilizer, it is admitted as an organic amendment in organic agriculture that can help to improve physical, chemical, and biological properties of soils (Wahid et al., 2016). Direct application of RP is suitable for acidic soils because RP solubilizes well in a low-pH environment, resulting in an increase of available P (Caravaca et al., 2004). However, in high-pH environments limited solubilization of RP strongly constrains P availability.

To effectively provide plant-available P through the application of RP, bio-inoculants, as individual species or as a consortium of microorganisms that are beneficial for plants, are added to release strongly sorbed or fixed P in RP *via* solubilization by organic acids and carboxylates and to subsequent acquire released P from the soil solution (Ahemad and Kibret, 2014). Among those types of bio-inoculants, phosphate solubilizing bacteria (PSB) and fungi (PSF), and arbuscular mycorrhizal fungi (AMF) are of interest due to their ability to desorb, solubilize and acquire P that is transferred to the plant, ultimately promoting plant growth and production (Fürnkranz et al., 2009; Hanif et al., 2015). PSB and PSF can directly provide available P to enhance plant growth even under drought (Nacoon et al., 2021a). Furthermore, some PSB and PSF also produce plant growth regulators or phytohormones such as indole acetic acid (IAA) (Etesami et al., 2015), gibberellins (GA) (Zhao and Zhang, 2015), proline (Vardharajula et al., 2011), 1-aminocyclopropane-1-carboxylate (ACC) deaminase, and exopolysaccharides (EPS) (Vurukonda et al., 2016). Because of these abilities and their environmentally friendly nature, PSB and PSF, therefore, can be an alternative to mineral-P fertilizers. Inoculation with AMF increases plant tolerance against various unfavourable conditions (Bowles et al., 2016; Abdel-Salam et al., 2018). Especially under drought, leaf number, chlorophyll concentration, stomatal conductance, rate of transpiration and photosynthesis were greater in mycorrhizal plants compared to non-mycorrhizal plants (Beltrano and Ronco, 2008; Abdelmoneim et al., 2014; Amico et al., 2016). Our previous research demonstrated that the AMF fungus *Rhizophagus aggregatus* (N.C. Schenck and G.S. Smith) C. Walker BM-3 g.3 improved drought tolerance in sunchoke as evidenced by increased root growth and plant yield (Nacoon et al., 2021a). An increase in available P has been found in the combined presence of PSB and AMF (Ordoñez et al., 2016; Boonlue et al., 2017; Nacoon et al., 2020; Nacoon et al., 2021b). This combined beneficial effect is due because PSB solubilize mineral P into available P, which is then acquired by AMF and transported to plants through AMF hyphae. Such combined effects are often called synergistic, however Larimer et al. (2014) outlined how to separate synergistic from additive effects from multiple symbionts. While their meta-analysis did not show synergistic effects between AMF and beneficial microbes involved in N_2_ fixation, they did not provide data whether interactions between AMF and PSB (or PSF) are synergistic or additive.

However, there is still limited knowledge on the combined effect of AMF and PSB on sunchoke under drought. Therefore, our study aimed to investigate the combined effects of PSB (*Burkholderia vietnamiensis* KKUT8-1) and AMF (*Rhizophagus aggregatus* BM-3 g.3) to enhance growth and yield of sunchoke under drought, in the presence and absence of RP. The findings in this work would provide a potential use of PSB and AMF as bio-inoculants for sustainable agriculture of sunchoke under drought in the future.

## 2 Materials and methods

### 2.1 Isolation and characterization of phosphate solubilizing bacteria

#### 2.1.1 Soil sample collection

Rhizosphere samples were collected from field-grown sunchoke at Agronomy farm in Khon Kaen University, Khon Kaen, Thailand (16.466371° N to 102.824° E). To obtain rhizosphere samples, soil samples were taken at a depth of 0 to 15 cm around plant roots, roots were separated from soil, and soil attached to roots was removed by shaking. Each sample was individually placed in an aseptic plastic bag and immediately stored in a cooler until arrival at the laboratory. The samples were stored at 4°C until isolation of the PSB.

#### 2.1.2 Evaluation of phosphate solubilization ability of PSB isolate

Phosphate solubilization ability of the isolates was investigated on Pikovskaya’s agar (PKV) medium containing 5% polyethylene glycol–6000 (PEG-6000). Single colonies were spot-inoculated on PKV agar medium and incubated at 35°C for 72 h. A clear zone (halo) around the colonies was indicative of positive phosphate solubilization. The efficiency of the PSB isolates to solubilize phosphate was evaluated based on the Phosphate Solubilization Index (PSI) as described by Morales et al. (2011). PSI was calculated as the ratio of the halo zone over the diameter of the colony.

#### 2.1.3 Quantitative determination of P solubilization by PSB

A 3 mL of starter inoculum (0.5 McFarland scale = 1x10^8^ CFU mL^-1^) was added to 30 mL of PKV medium mixed with different concentrations of PEG–6000 (0, 10, 20 and 30%). The cultures were incubated with shaking at 150 rpm at 35°C for 72 h. After that, pH of the cultures was determined prior to centrifugation at 12,000 rpm for 2 min to remove bacterial cells. The concentration of available P was determined according Murphy and Riley (1962) with some modification. Briefly, 2 mL of sample supernatant was mixed with 5 mL of 2% boric acid, 2 mL of Murphy’s reagent and 1 mL of 2.5% ascorbic acid. The absorbance of the mixture was measured at a wavelength of 820 nm using a spectrophotometer (U-5100, Hitachi, Japan). The quantities of available P in the samples were determined by comparison with the KH_2_PO_4_ standard curve.

#### 2.1.4 Identification of PSB isolate

The PSB isolate exhibiting the highest P solubilization activity based on highest available P concentrations at high osmotic stress (**Supplementary Table 1**). KKUT 8-1 was identified by 16S rDNA sequence analysis. Genomic DNA extraction was carried out from cells grown in nutrition broth for 18 h using a modified protocol described by Sarikhani et al. (2018). Then, PCR amplification of 16S rDNA sequence was carried out using primer pair 1525R (5’-AAAGGAGGTGATCCAGCC-3’) and 27F (5’-AGAGTTTGATCCTGGCTCAG-3’). The reaction condition included an initial denaturation of 4 min at 95°C, followed by 30 cycles of denaturation at 95°C for 30 seconds, annealing at 55°C for 45 seconds, and extension at 72°C for 1 min. A final extension at 72°C for 10 min was carried out at the end of amplification. The PCR product was purified using a GeneJET PCR purification Kit (Thermo Scientific, Lithuania) prior to submitting for sequencing at the 1st Base Laboratories Sdn Bhd, Malaysia. The 16S rDNA sequence analysis was performed using MEGA7 software (Kumar et al., 2016) and compared with sequences in GenBank on the NCBI database.

#### 2.1.5 Determination of indole-3 acetic acid (IAA) production

The culture of KKUT8-1 was grown in nutrient broth and then incubated with shaking at 150 rpm at 40°C for 28 h. After incubation, the culture was centrifuged at 12,000 rpm for 2 min to retrieve supernatant. Then, 1 mL of supernatant was mixed with 2 mL of Salkowski’s reagent modified from Akter and Rahman (2013) (35% Perchloric acid and 0.5M FeCl_3_.6H_2_O; in volume of 98 mL: 2 mL (v/v)). The mixed reagent was incubated under dark conditions for 30 min. Then, the absorbance of the mixture was measured at a wavelength of 530 nm using a spectrophotometer (U-5100, Hitachi, Japan). The quantities of IAA in the samples were determined by comparison with the IAA standard curve.

#### 2.1.6 Determination of proline production

The culture of KKUT8-1 was incubated with shaking at 150 rpm at 40°C for 28 h. Proline concentration was determined both intracellularly and extracellularly according to Bates et al. (1973). To determine extracellular proline, the bacterial culture was centrifuged at 8,000 rpm for 10 min to remove cell pellets. The supernatant was kept for proline determination. Thereafter, to determine intracellular proline production, cell pellets were washed using 0.85% normal saline solution prior to proline extraction using 3% sulfosalicylic acid. The mixture was filtered through a filter paper Whatman No.1 in order to collect the filtrate for proline quantification. To quantify proline concentration, 2 µL of samples were mixed with 2 mL of acid ninhydrin and 2 mL of glacial acetic acid. The mixture was boiled at 100°C for 1 h, then the tube was immediately transferred into an ice bath in order to stop the reaction. Proline in the solution was extracted using 4 mL of toluene and the solution was vigorously vortexed for 15–20 seconds. The absorbance at a wavelength of 520 nm was measured by using toluene as a blank. Proline concentration was calculated by extrapolating with a standard curve of proline.

#### 2.1.7 Determination of ACC deaminase production

ACC deaminase analysis method was modified from Ali et al. (2014) and Namwongsa et al. (2019). The culture of KKUT8-1 was incubated at 40°C for 28 h. Cell pellets were collected by centrifugation and then washed twice using 0.1 M Tris–HCl (pH 7.5). Cell pellets were separated into 2 flasks containing 2 mL of Dworkin and Foster medium and 2 mL of 3 mM 1–aminocyclopropane–1–carboxylic acid (ACC) each flask. One flask was added with 20% PEG-6000 whereas the other was left without PEG-6000. Then, the mixture was incubated with shaking at 150 rpm at 40°C for 28 h. After incubation, cell pellets were collected by centrifugation at 5,000 rpm for 5 min, and then washed twice with 0.1 M Tris–HCl (pH 7.5) prior to resuspending in 200 μL of 0.1 M Tris–HCl (pH 8.5). The cell suspension was vortexed with 5% (v/v) toluene at the highest speed for 30 seconds. Then, 50 μL of cell suspension was mixed with 5 μL of 0.3 M ACC and incubated at 30°C for 30 min. The tube containing 50 μL of cell suspension without ACC was set up as a negative control. A mixture of 50 μL of 0.1 M Tris–HCl (pH 8.5) and 5 μL of 0.3 M ACC was used as a blank solution for spectrophotometric measurement. The samples, negative control and a blank were mixed thoroughly with 500 μL of 0.56 N HCl using a vortex mixer. Cell debris was removed by centrifugation at 12,000 g for 5 min. A 500 μL aliquot of the supernatant was transferred to a glass test tube and mixed with 400 μL of 0.56 N HCl and 150 μL of DNF solution (0.1 g of 2,4–dinitrophenylhydrazine in 100 mL of 2 N HCl). The mixture was then incubated at 30°C for 30 min. One mL of 2N NaOH was added to the sample before the absorbance at 540 nm was measured. ACC deaminase activity was calculated by comparing with a standard curve of α–ketobutyrate.

#### 2.1.8 Phosphatase activity analysis

Phosphatase activity analysis was modified from the method of Behera et al. (2017a) and Zhao and Zhang (2015). The culture of KKUT8-1 was incubated at 40 °C for 24, 48 and 72 h. After incubation, the cultures were centrifuged at 4°C, 8,000 rpm for 10 min to collect supernatant. Alkaline and acid phosphatase activities were analysed by using *p*-nitrophenyl phosphate (pNPP) as a substrate, which was then transformed into a coloured product of *p*-nitrophenol (pNP). One mL of supernatant was mixed with 4 mL of 0.1 M citrate buffer pH 5.0 and 0.1 M carbonate buffer pH 9.7 for the analysis of acid and alkaline phosphatases, respectively. Then, 1 mL of 0.025 mM of pNPP was added and the mixtures were incubated at 37°C for 1 h. Thereafter, 0.2 mL of toluene was added prior to stopping the reaction by adding 4 mL of 0.5 M NaOH and 1 mL of 0.5 M CaCl_2_. The mixtures were centrifuged at 5,000 rpm for 10 min. The absorbance at a wavelength of 420 nm was measured using a spectrophotometer and the concentrations of PNP in the samples were determined by comparing with the standard curve of PNP. One unit of acid or alkaline phosphatase was defined as the amount of enzyme activity that liberates 1 µmoL of PNP in 1 min under a standard condition (pH 5.0 for acid phosphatase or 9.7 for alkaline phosphatase, at 37°C).

#### 2.1.9 Determination of organic acid production

To determine organic-acid production at different temperatures, the isolate KKUT8-1 was grown in Pikovskaya’s broth medium with and without 20% PEG–6000 and incubated with shaking at 150 rpm at 40°C for 28 h. Then, the cultures were centrifuged at 4°C, 8,000 rpm for 10 min to remove cell pellets. The supernatant was filtered through a syringe filter (0.2 µm pore size diameter) prior to high-performance liquid chromatography (HPLC) analysis. The 20 µL of filtrate were injected into a HPLC column LC–20AD (Shimadzu, Kyoto, Japan) equipped with a SPD–M20A diode array detector. 10 mM KH_2_PO_4_ in phosphoric acid (pH 2.7) was used as a mobile phase. The signals were recorded at a wavelength of 210 nm. The concentrations of organic acids including citric, gluconic, lactic, maleic, malic, malonic, succinic, propionic, and tartaric acids in the samples were determined by comparing with the peak areas of their corresponding standards.

#### 2.1.10 Determination of exopolysaccharide production

Determination of EPS production was carried out as described by Ali et al. (2014). The culture of KKUT8-1 was grown in nutrient broth supplemented with and without 20% PEG-6000 and then incubated with shaking at 150 rpm at 40°C for 28 h. After incubation, the cultures were centrifuged at 4,000 rpm for 10 min to collect supernatant. The supernatant was diluted with 10 mL of 0.85% potassium chloride (KCl) solution to reduce viscosity before centrifugation. The supernatant was filtered through filter paper (Whatman No. 1) to collect filtrate for dialysis against sterile distilled water at 4°C. The dialysate was centrifuged to remove any insoluble materials prior to mixing with 3 volumes of ice-cold absolute alcohol. The mixture solution was kept at 4°C overnight to precipitate EPS. Centrifugation at 4,000 rpm for 10 min was carried out to retrieve precipitated EPS. Further purification of EPS was performed by repeating the dialysis and precipitation steps. Total carbohydrate concentration in the precipitated EPS was determined according to Dubois et al. (1956).

### 2.2 Application of AMF and PSB for promoting growth of sunchoke under drought

#### 2.2.1 Soil preparation

Soils were air-dried and then sieved through a 2 mm-mesh diameter sieve. Physicochemical properties of soils were as follows; loamy sand type, pH 6.78, electrical conductivity 0.050 dS m^-1^, cation exchange capacity 2.37 cmol ^(+)^ kg^-1^ and organic matter concentration 3.65 g kg^-1^. Soil nutrient composition was as follows: total N 0.29 g kg^-1^, total P 20.5 mg kg^-1^, total K 392.8 mg kg^-1^, available P, K, Ca and Na were 8.4, 30.6, 277.8 and 27.7 mg kg^-1^, respectively. Bulk density was 1.6 g cm^-3^, the permanent wilting point (PWP) 2.7% and the field capacity (FC) 11.5%. The soil was packed into a pot (38-cm diameter, 28-cm depth) divided into 2 layers, in which the first layer, containing 15 kg of soil was filled into the bottom layer to have the height of 5 cm away from the pot mouth. Then, a drip line was installed and covered with 5 kg of soil as top layer. Plastic tubes were connected with the drip line and used to supply water to the surface soil. Water was supplied at field capacity one day before planting.

#### 2.2.2 Water management

The water deficit level of this experiment was slightly modified by Ruttanaprasert et al. (2016); Namwongsa et al. (2019); Nacoon et al. (2021a). Two different watering systems were set up as follows: 1.) the well–watered treatment (WW) in which the field capacity was maintained uniformly since transplanting until harvesting and 2.) the drought treatment (DS) in which the water supply was reduced to 1/3 of available water (AW) 20 days after transplantation (DAT) until harvesting. Soil moisture content was maintained uniformly with no more than 1% variance. The amount of water needed was calculated following Doorenbos and Pruitt (1977). Soil moisture in the WW condition was AW 11.5%, while that of the DS condition was 5.6%.

#### 2.2.3 Experimental design

The experiment was set up in the greenhouse of Khon Kaen University’s agronomy farm in Khon Kaen, Thailand (16° 28′N, 102° 48′E, 200 meters above sea level). A four-factorial experiment with a Randomized Complete Block Design (RCBD) with four replications was conducted in the greenhouse. The factors investigated were: (1) PSB (presence or absence of bacterium KKUT8-1); (2) AMF (presence or absence of *Rhizophagus aggregatus*; (3) water regime (WW = field capacity and DS = 1/3 of available water); (4) RP (without or with 6-gram RP per pot). RP contained 25% P_2_O_5_ as total P.

#### 2.2.4 Inoculation with PSB and AMF

The cultures of an AMF (*Glomus aggregatum* BM-3 g.3) and a PSB (KKUT8-1, which was molecularly identified as *Burkholderia vietnamiensis*) were obtained from the Microbiology laboratory, Department of Microbiology, Faculty of Science, Khon Kaen University, Thailand. KKUT8-1 culture was incubated with shaking at 150 rpm, 30°C for 24 h. The culture was centrifuged at 6,000 rpm for 15 min and then washed twice with 0.85% NaCl. Prior to planting, 27 g of AMF soil inoculum was applied to each plant at a depth of 5 cm, resulting in a starting AMF inoculum concentration of 75 spores g^-1^ soil. After that, a single plant seedling was transferred into the hole where the AMF inoculum was added (2,000 spores per plant). Then, 10 mL of PSB inoculum (1x10^9^ CFU mL^-1^ per seedling) was poured onto the seedlings adjacent to the roots. No microbial wash was applied to the treatments without inoculation of AMF or PSB.

#### 2.2.5 Seedling preparation

Sunchoke seedlings (cv. HEL65) were prepared according to Ruttanaprasert et al. (2014). Briefly, tubers were cut into small pieces with two to three buds per piece. These tuber pieces were pre-sprouted in a coconut peat medium under ambient conditions for 4 to 7 days. They were then transferred to germinating plug trays containing a mixed medium of charred rice husks and soil. The tuber pieces were left to germinate for 7 days until complete sprouting. Uniform and healthy seedlings were transplanted to the pot experiment.

#### 2.2.6 Plant growth and physiological analysis

At 60 DAT, plant growth parameters were assessed. SPAD values were measured using a SPAD 502-plus (Konica Minolta, Japan). Leaf area index (LAI) was determined using a LI-3100C area meter (LI-COR Bioscience). The first fully expanded leaves from the top of each pot were analysed for photosynthetic rate, stomatal conductance, and transpiration rate using a LI-6400XT portable photosynthesis system (LI-COR Bioscience). Water use efficiency (WUE) was calculated by dividing photosynthetic rate by transpiration rate. Fresh roots were collected and scanned using a root scanner (Epson perfection V700 photo) and then analysed for root length, root diameter, root volume and root surface area using WinRhizo. Total biomass of roots, stem and leaves was determined after drying at 80°C for 3 days. We also determined the number of tubers per plant, tuber fresh and dry weight. Inulin concentration was determined following the method described by Saengkanuk et al. (2011). Dried stems and leaves were used for determining P concentration after wet oxidation by mixing with nitric acid and perchloric acid. Total P concentration was determined using the molybdovanadate with acid persulfate digestion method (Twine and Williams, 1971), and then the absorbance of the samples was measured using a spectrophotometer at a wavelength of 420 nm.

#### 2.2.7 Determination of relative water content, total chlorophyll concentration and proline concentration

Relative water content (RWC) was determined according to Janket et al. (2013). Leaves were cut into 1 cm-diameter pieces using a disc borer, and then leaf fresh weight (FW) was determined. The leaf discs were placed in distilled water until saturated with moisture under darkness condition for 8 hours and the turgid weight (TW) was determined. The leaf discs were oven-dried at 80°C for 48 hours prior to determination of leaf dry weight (DW). Water content was calculated according to Kramer (1980):


RWC = [(FW–DW)/(TW–DW)]×100

Chlorophyll concentration was determined from 100 mg of fresh leaves soaked in 25 mL acetone (80%) in the dark at room temperature. The absorbance solution was measured at a wavelength of 470, 646 and 663 nm using a UV/Vis spectrophotometer (Li et al., 2020). Determination of proline was carried out following Claussen (2005). Proline was extracted from leaf samples using 3% sulfosalicylic acid at 100°C for 10 min. The mixture was filtered through filter paper to retrieve filtrate. Two mL of filtrate was mixed with 2 mL ninhydrin solution and 2 mL acetic acid and then incubated at 100°C for 30 min. The coloured product formed was extracted by mixing with 4 mL toluene and vigorously shaken. The absorbance of the resultant material was measured at a wavelength of 520 nm.

#### 2.2.8 Quantification of total soluble sugar and malondialdehyde concentration

Total soluble sugars (TSS) were determined using the anthrone method according to Irigoyen et al. (1992). TSS concentration was measured from fresh leaf tissue (0.1 g), homogenized with 4 mL ethanol (80%). 0.5 mL ethanol extract was mixed with 3 ml of freshly-prepared anthrone solution in the tube (200 mg anthrone + 100 ml of 72% (w/w) H_2_SO_4_), and then placed in a boiling water bath (100°C) for 10 min. The solution was left to cool at room temperature before measuring absorbance at a wavelength of 620 nm using a spectrophotometer. The TSS concentration was calculated by comparing with a standard curve of glucose and expressed in terms of mg g plant^-1^. To determine malondialdehyde (MDA) concentration, 0.1 g of leaves tissues were macerated in 10% (w/v) trichloroacetic acid solution (TCA). Samples were vortexed and then centrifuged at 4,000 rpm for 10 min to collect supernatant. Two mL of the solution containing 0.6% (w/v) of thiobarbituric acid (TBA) dissolved in 10% TCA solution was added to 2 mL of sample supernatant. The mixture was incubated at 100°C for 20 min and then immediately cooled in an ice bath to stop the reaction. After that, the reaction mixture was centrifuged at 10,000 rpm for 5 min to retrieve supernatant. The absorbance at 450, 532 and 600 nm was measured using a spectrophotometer. MDA concentration was calculated according to Boutasknit et al. (2020):


MDA (M g– 1) = [6.45(OD532 – OD600)] – (0.56×OD450)

#### 2.2.9 Phosphatase assay in soil

Acid and alkaline phosphatase in soil were determined according to Majumder and Das (2016). One gram of fresh soil was mixed with 0.25 mL of toluene and 1 mL of 0.1 M sodium citrate buffer pH 5.0 (for acid phosphatase) or 0.1 M sodium citrate buffer pH 9.7 (for alkaline phosphatase). One mL of pNPP solution was added and the solution was vigorously vortexed. The mixture was incubated at 37°C for 40 min. Analysis of phosphatase activity was performed as described previously.

#### 2.2.10 Electrolyte leakage

Electrolyte leakage (EL) was determined from leaf discs 1.0 cm in diameter excised from the lowest leaves according to the modified methods of Zolfaghari et al. (2020) and Jungklang et al. (2017). Each leaf disc was rinsed twice with deionized water for 2–3 min. Then, 8 pieces were added in 7 mL of deionized water in test tubes and shaken at 25°C for 22 h prior to determination of electrical conductivity (EC_1_). Conductivity of the leaf solution was measured using a conductivity meter (Mettler Toledo AG, 8603 Schwerzenbach, Switzerland). The solution was boiled for 30 min to achieve 100% electrolyte leakage before measuring total conductivity (EC_2_). Electrolyte leakage was expressed as a percentage of total conductivity according to:


EL (%) = (EC1/EC2) × 100

#### 2.2.11 Assessment of mycorrhizal root colonization and PSB population size

Plant roots were stained following a modified method described by Koske and Gemma (1989). Fractional mycorrhizal root colonization was estimated by the method described by Trouvelot et al. (1986). The enumeration of PSB was done by the dilution plate count technique on Pikovskaya’s agar medium. The experiment was carried out in triplicate. Plates were incubated at 30°C for 3 days. The colonies that showed a clear halo zone indicating P solubilization were counted (Minaxi et al., 2013; Rahimzadeh and Pirzad, 2017a).

### 2.3 Statistical analysis

Data were subjected to analysis of variance (ANOVA) using Statistix10 software, after testing for normality and homogeneity of variances. Multiple comparisons of ANOVA with least significant difference (LSD) at P<0.05 was performed Correlations among plant growth parameters, AMF colonization, PSB population and soil moisture were investigated using correlations (Pearson’s correlation coefficient) and evaluated at a significance level of P< 0.05.

## 3 Results

### 3.1 Isolation and characterization of phosphate solubilizing bacteria

#### 3.1.1 Qualitative determination of P solubilization of PSB

Phosphate solubilizing ability was recorded in 10 out of 40 bacterial isolates that exhibited a halo zone surrounding their colonies. Phosphate solubilizing index (PSI) of those isolates ranged from 0.95 to 2.50. The highest value of PSI was found in isolate KKU-8.3, while isolate KKU-23 showed the lowest PSI value (**Supplementary Table 1**). The table also shows that 8 out of 10 PSB isolates solubilized P both in the presence and absence of PEG. Concentrations of available P decreased with increasing concentrations of PEG-6000, indicating that the presence of PEG-6000 negatively affected phosphate solubilization activity. Note that the condition with 20% PEG resembles drought under the greenhouse experiment, so the PSB isolate capable of releasing high amounts of P under 20% and 30% PEG (KKUT8-1) was considered the best P-solubilizing bacterium and selected for species identification and further use in the greenhouse experiment. Its 16S rDNA sequence (1,400 bp) was closely related to the sequence of *Burkholderia vietnamiensis* with 98% identity. Therefore, this isolate was designated as *Burkholderia vietnamiensis* strain KKUT8-1. In order to ensure its non-pathogenicity, a single colony was cultured on a blood agar plate and incubated at 37 °C for 24 hr. The results showed that there was no clear zone around the colonies, indicating the absence of haemolytic activity (data not shown). Therefore, strain KKUT8-1 was not considered as a human pathogen.

#### 3.1.2 Phosphatase activity, and production of organic acid, IAA, proline, ACC deaminase, and exopolysaccharide by KKUT8-1

**Figure 1:**
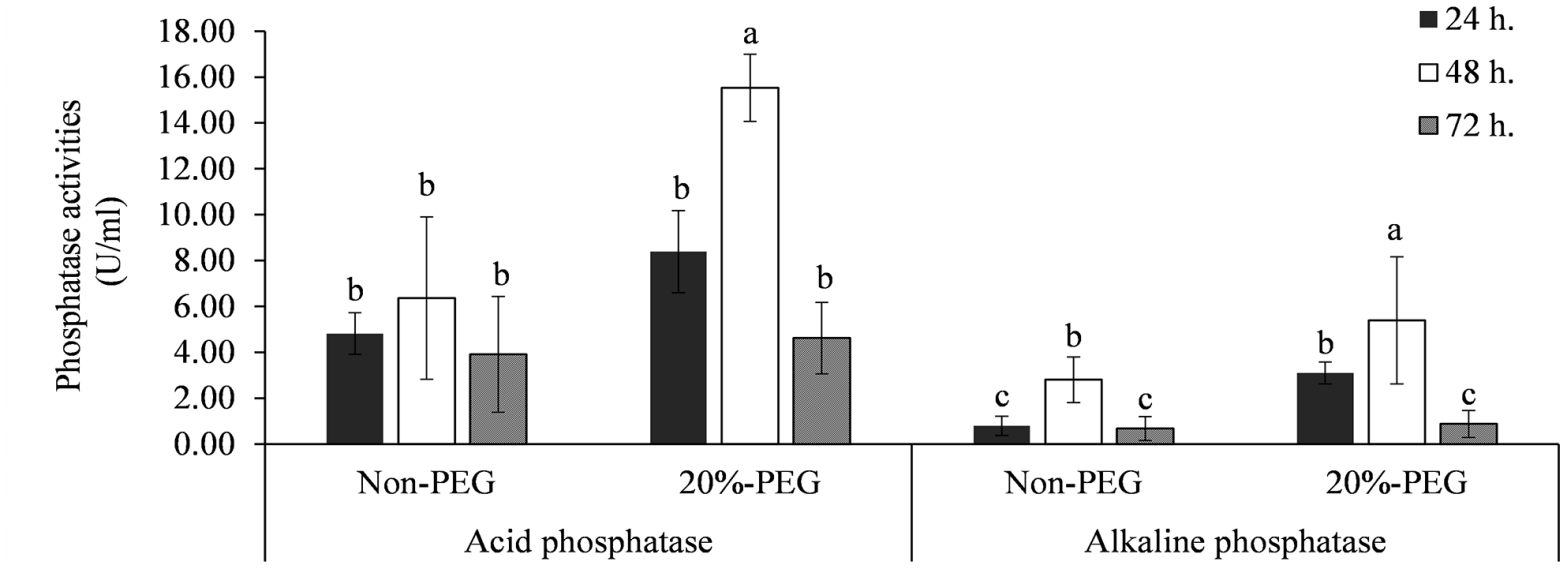
Enzyme activities of acid and alkaline phosphatases produced by KKUT8-1 in the cultures containing 0 and 20% of PEG during 72 h of incubation. Different letters indicate significant differences at P <0.05 by LSD.

Strain KKUT8-1 exhibited both acid and alkaline phosphatase activities (**Figure 1**). The highest activity of acid phosphatase of 15.5 U mL^-1^ and alkaline phosphatase of 5.4 U ml^-1^ was found with 20% PEG., suggesting that osmotic stress induced an increase in phosphatase production. Highest phosphatase production was recorded after 48 hours.

HPLC analysis of the culture filtrates of KKUT8-1 showed the presence of multiple organic acids (**Table 1**), with gluconic, tartaric, D- and L-malic, malonic, lactic, maleic, citric and succinic acids being produced under both 0% and 20% PEG in comparable amounts. IAA production was much higher (160 μg g^-1^) in the absence of 20% PEG than in its presence (30 μg g^-1^; **Table 1**). Proline was produced both in the presence and absence of 20% PEG, with higher amounts of extracellular proline than intracellular proline. But in the presence of 20% PEG relatively more proline was retained intracellularly (**Table 1**). KKUT8-1 produced ACC deaminase both in the presence and absence of PEG, with higher amounts in the presence of 20% PEG (0.0350 μM h^-1^) than in its absence (0.0145 μM h^-1^; **Table 1**). KKUT8-1 produced high concentration of glucose (17.07 μg g^-1^) in the presence of 20% PEG (**Table 1**). Therefore, its ability to solubilize P and produce plant growth promoting substances (phosphatases, organic acids, IAA, proline, ACC deaminase and EPS) under osmotic stress, indicates KKUT8-1 to be a promising plant growth promoter under drought.

**Table 1:** Total concentrations of organic acids, indole acetic acid, proline, α-ketobutyrate (ACC deaminase determination), glucose (EPS determination) and type of organic acids produced by strain KKUT8-1 grown without PEG and with 20% PEG incubated at 40°C.

PEGconcentration	Type of organic acid	Total organic acids concentration(μg g^-1^)	IAA concentration(μg g^-1^)	Proline concentration(μg g^-1^)		α-ketobutyrate concentration (µM h^-1^)	Glucose concentration(μg g^-1^)
				Cell pellet(Intracellular)	Supernatant(Extracellular)		
0%	gluconic, tartaric, D- and L-malic, malonic, lactic, maleic, citric, succinic acid	5,584	160	12.3	2.8	0.015	11.4
20%	gluconic, tartaric, D- and L-malic, malonic, lactic, maleic, citric, succinic acid	5,411	30	17.2	1.6	0.035	17.1

### 3.2 Application of AMF and PSB for promoting growth of sunchoke under drought

Plant performance parameters were determined at 60 DAT. ANOVA results are presented in **Table 2**. For aboveground biomass AMF, water and the AMF × water interactions were significant sources of variation, whereas PSB and RP, and the other interactions were not. Leaf area exhibited the same general pattern. SPAD values were significantly affected by AMF, PSB and the interaction AMF × PSB, whereas water and RP and the other interactions were not significant sources of variation. Inulin concentration was significantly affected by AMF, PSB, and the interactions AMF × PSB, PSB × RP, and the three-way interactions AMF × PSB × RP. Photosynthesis rate (Pn), water use efficiency (WUE), stomatal conductance (SC) and transpiration rate (Tr) were always affected by water. AMF was a significant source of variation for three gas-exchange parameters (except photosynthesis rate), whereas for photosynthesis rate RP was a significant source of variation. PSB was not a significant source of variation (**Table 2**). A few interaction terms were also significant sources of variation, but these did not exhibit a consistent pattern. The effect of AMF, PSB, water and RP on root traits are presented in **Table 3**. Both AMF and water where significant sources of variation, whereas the other main factors and all interactions were not.

**Table 2:** Plant performance of sunchoke treated with AMF and PSB under well-watered (WW) and drought (DS) conditions, in the presence and absence or RP, evaluated at 60 DAT.

Waterconditions	Treatments	Above ground plant biomass (g)	Leafarea(cm^2^)	SPADvalue	Number of tubers	Tuber dry weight(g)	Inulin concentration (%)	Pn(µmol CO_2_ m^-2^ s^-1^)	WUE(µmol CO_2_/H_2_O m^-2^s^-1^)	SC(H_2_O m^-2^s^-1^)	Tr(mmol H_2_O m^-2^s^-1^)
Well- watered	Control	4.8 e	241 cd	35.3 e	2.5 d	3.4 cd	29.0 f	22.5 b-e	7.1 de	0.36 abc	3.1 abc
	AMF	18.3 a	717 a	46.5 a	5.8 ab	7.5 a	39.6 ab	24.5 abc	8.7 b-e	0.30 b-e	2.9 a-e
	PSB	5.1 e	319 c	41.1 d	2.0 d	2.9 cd	34.7 b-e	27.0 a	8.4 cde	0.45 a	3.3 a
	AMF+PSB	15.9 ab	748 a	45.1abc	4.8 bc	5.2 bc	35.3 b-e	22.6 b-e	9.8 a-d	0.27 b-e	2.5 b-f
	RP	5.9 e	255 cd	34.1 e	2.5 d	3.6 cd	27.9 f	21.6 cde	10.8 abc	0.31 b-e	2.1 ef
	AMF+RP	16.4 ab	7056 a	45.1 abc	4.8 bc	6.3 ab	34.1 cde	20.8 de	8.5 cde	0.24 cde	2.5 a-f
	PSB+RP	4.8 e	167 d	42.6 bcd	3.3 cd	3.2 cd	37.4 bcd	24.1 a-d	7.6 de	0.36 abc	3.2 ab
	AMF+PSB+RP	15.5 bc	729 a	44.5 a-d	5.3 ab	7.8 a	38.8 abc	22.8 b-e	9.5 a-d	0.27 cde	2.5 b-f
Drought	Control	4.7 e	214 cd	34.8 e	2.5 d	2.5 d	31.7 ef	22.8 b-e	7.4 de	0.40 ab	3.0 a-d
	AMF	11.0 d	488 b	45.0 abc	2.8 d	3.0 cd	35.4 b-e	20.3 ef	11.6 ab	0.18 e	1.8 f
	PSB	4.7 e	271 cd	42.0 cd	2.8 d	2.1 d	38.4 abc	25.7 ab	11.6 ab	0.28 b-e	2.4 c-f
	AMF+PSB	12.4 d	491 b	47.0 a	4.5 bc	5.1 bc	34.2 cde	21.8 cde	10.1 a-d	0.22 de	2.2 def
	RP	3.4 e	206 d	32.1 e	2.0 d	2.3 d	32.5 def	19.6 ef	6.1 e	0.36 abc	3.3 ab
	AMF+RP	13.0 cd	538 b	45.3 abc	6.5 a	5.0 bc	35.0 b-e	22.8 b-e	12.1 a	0.23 de	2.0 ef
	PSB+RP	3.8 e	217 cd	42.0 cd	2.0 d	1.9 d	36.6 b-e	17.2 f	11.6 ab	0.18 e	1.8 f
	AMF+PSB+RP	11.4 d	507 b	46.1 ab	5.5 ab	4.8 bc	43.1 a	20.9 de	10.8 abc	0.20 de	2.1 ef
Four-way ANOVA											
	AMF	**	**	**	**	**	**	ns	*	**	**
	PSB	ns	ns	**	ns	ns	**	ns	ns	ns	ns
	Water	**	**	ns	ns	**	ns	**	**	**	**
	RP	ns	ns	ns	ns	ns	ns	**	ns	ns	ns
	AMF × PSB	ns	ns	**	ns	ns	*	ns	ns	ns	ns
	AMF × water	**	**	ns	ns	ns	ns	ns	ns	ns	ns
	AMF × RP	ns	ns	ns	ns	ns	ns	**	ns	ns	ns
	PSB × water	ns	ns	ns	ns	ns	ns	ns	ns	ns	*
	PSB × RP	ns	ns	ns	ns	ns	**	ns	ns	ns	ns
	Water × RP	ns	ns	ns	ns	ns	ns	ns	ns	ns	ns
	AMF × PSB × water	ns	ns	ns	ns	ns	ns	ns	**	*	**
	AMF × PSB × RP	ns	ns	ns	ns	ns	*	ns	ns	ns	ns
	AMF × water × RP	ns	ns	ns	**	ns	ns	*	ns	ns	ns
	PSB × water × RP	ns	ns	ns	*	*	ns	*	ns	ns	*
	AMF × PSB × water × RP	ns	ns	ns	ns	ns	ns	ns	ns	ns	ns

**, Significant difference at P< 0.01; *, Significant difference at P< 0.05; ns, non-significant difference. Data are the means of three replications. Values with similar lowercase letters in each column are not significantly different according to LSD at P< 0.05. Pn, Photosynthetic rate; WUE, Water use efficiency; SC, Stomatal conductance; Tr, Transpiration rate.

**Table 3:** Root growth performance of sunchoke treated with AMF and PSB under well-watered (WW) and drought (DS) conditions, in the presence and absence or RP, evaluated at 60 DAT.

Water conditions	Treatments	Root length(cm)	Root surfaceArea (cm^2^)	Root diameter(mm)	Root volume(cm^3^)	Specific root length(m/g)	Root tissue density (g/cm^3^)
Well- watered	Control	2363.0 d	454.9 fg	0.34 ef	5.30 fgh	16.098 c-f	0.6843 ab
	AMF	14510.0 a	1593.2 a	0.39 a-d	16.17 a	23.22 abc	0.372 d
	PSB	3943.0 d	791.0 de	0.35 def	6.90 ef	27.993 a	0.4898 bcd
	AMF+PSB	11079.0 bc	1314.7 abc	0.39 a-d	12.97 bc	22.553 a-d	0.379 d
	RP	3956.0 d	753.9 def	0.34 f	6.46 fg	16.625 b-f	0.7153 ab
	AMF+RP	13604.0 ab	1188.1 bc	0.41 ab	12.25 bcd	24.82 a	0.3123 d
	PSB+RP	1820.0 d	780.4 de	0.37 b-f	6.00 fgh	14.155 ef	0.6825 ab
	AMF+PSB+RP	14180.0 a	1405.1 ab	0.42 a	14.63ab	23.595 ab	0.3188 d
Drought	Control	2374.0 d	576.7 efg	0.37 b-f	5.49 fgh	15.638 def	0.6415 abc
	AMF	8665.0 c	1019.8 cd	0.40 abc	10.20 cd	21.865 a-d	0.3695 d
	PSB	3475.0 d	595.4 efg	0.40 abc	3.70 gh	26.615 a	0.3042 d
	AMF+PSB	10183.0 c	1120.3 bc	0.39 a-d	10.39 cd	21.365 a-e	0.4033 d
	RP	2315.0 d	371.5 g	0.36 c-f	3.09 h	13.355 f	0.8305 a
	AMF+RP	10519.0 c	1196.7 bc	0.39 a-d	11.74 bcd	21.55 a-d	0.4013 d
	PSB+RP	1427.0 d	444.8 g	0.38 a-e	3.50 h	13.978 f	0.67 ab
	AMF+PSB+RP	8276.0 c	1012.1 cd	0.38 a-f	9.64 de	21.517 a-d	0.4147 cd
Four-way ANOVA							
	AMF	**	**	**	**	**	**
	PSB	ns	ns	ns	ns	ns	*
	water	**	**	ns	**	ns	ns
	RP	ns	ns	ns	ns	ns	*
	AMF x PSB	ns	ns	ns	ns	*	*
	AMF x water	**	ns	**	ns	ns	ns
	AMF x RP	ns	ns	ns	ns	**	*
	PSB x water	ns	ns	ns	ns	ns	ns
	PSB x RP	ns	ns	ns	ns	*	ns
	Water x RP	ns	ns	ns	ns	ns	ns
	AMF x PSB x water	ns	ns	ns	ns	ns	ns
	AMF x PSB x RP	ns	ns	ns	ns	*	ns
	AMF x water x RP	ns	*	ns	ns	ns	ns
	PSB x water x RP	ns	ns	ns	ns	ns	ns
	AMF x PSB x water x RP	*	*	ns	**	ns	ns

**, Significant difference at P< 0.01; *, Significant difference at P< 0.05; ns, non-significant difference. Data are the means of three replications. Values with similar lowercase letters in each column are not significantly different according to LSD at P< 0.05.

**Figure 2:**
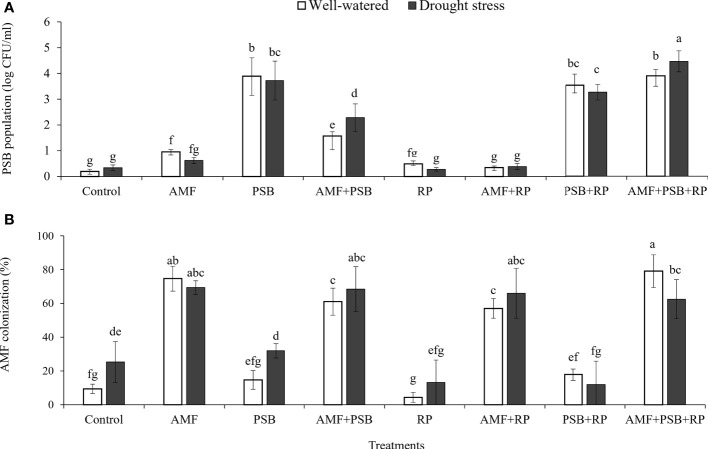
PSB population in rhizosphere soil **(A)** and percentage of AMF colonization in plant roots **(B)** under well-watered and drought conditions evaluated at 60 DAT. Different letters indicate significant differences at P<0.05 by LSD. Treatment means are the average of four replications.

AMF had a positive effect on plant biomass, leaf area, SPAD values, number and dry weight of tubers, inulin concentration, and most gas exchange parameters. whereas PSB had a positive effect on SPAD values and inulin concentration, and water on plant biomass, leaf area, root length and root surface area (**Table 3**). The application of RP did not significantly improve plant performance apart from a negative effect on photosynthetic rate. Our data indicate that improved growth performance of sunchoke depended largely on the application of AMF and/or PSB. The significant interaction between AMF and water for plant biomass and leaf area indicates that mycorrhizal plants had a larger beneficial effect under drought. There was evidence for synergism between AMF and PSB for SPAD, as evidenced by the significant interaction term, but for most other parameters there was no evidence for synergy. Mycorrhizal plants had a larger root system than plants on the non-inoculated plants, and well-watered plants had a larger root system than plants grown under drought. AMF-inoculated plants also produced thicker roots than plants that were not-inoculated with AMF (**Table 3**). As the experiment was not executed under sterile conditions, plants in the treatment without AMF inoculum were also colonized by AMF, but colonization levels were significantly lower than those of plants that received AMF inoculum (16% versus 67%; **Figure 2**). Similarly, the PSB populations were significantly higher in treatments where they had been added, with a further enhancement of their population size after addition of RP (**Figure 2**).

**Figure 3:**
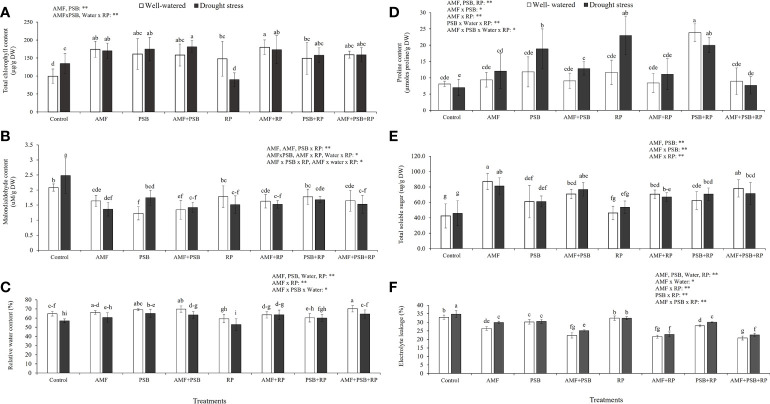
Physiological responses of sunchoke inoculated with AMF and PSB under well-watered (WW) and drought (DS) conditions evaluated at 60 DAT. **(A)** Total chlorophyll concentration, **(B)** Malondialdehyde concentration, **(C)** Relative water content (RWC), **(D)** Proline concentration, **(E)** Total soluble sugar concentration (TSS) and **(F)** Percentage of electrolyte leakage (%EL). Different letters indicate significant differences at P< 0.05 by LSD. Treatment means are the average of four replications. The significant effects of AMF, water, PSB, RP and their interactions are presented as the F value with test of significance (*P< 0.05; **P< 0.01) of four-way ANOVA. Non-significant factors are not shown.

**Figure 3** shows total chlorophyll concentration, MDA concentration, RWC, proline concentration, TSS concentration and %EL from plant leaves. The results indicated that AMF, PSB, and often the AMF × PSB and PSB × RP were significant sources of variation, whereas water was not. Both AMF and PSB resulted in increases in chlorophyll concentration (**Figure 3A**). Both AMF and PSB resulted in lower malondialdehyde concentrations compared to the control (**Figure 3B**), consistent with reduced plant stress after inoculation. Furthermore, the relative water content (RWC) of plants was significantly affected by all four factors, being significantly negatively affected by drought, and positively by inoculation with AMF and PSB (**Figure 3C**). Proline concentration was higher as a consequence of treatments with AMF, PSB or RP (**Figure 3D**). Total soluble sugar (TSS) was significantly affected by AMF, PSB and the interaction AMF × PSB, whereas water was not a significant source of variation. The presence of AMF and PSB resulted in higher amounts of total soluble sugars (**Figure 3E**). Electrolyte leakage was significantly affected by all four main factors and several interaction terms. The presence of microbial inoculum, water, and RP resulted in reduced leakage compared to the control (**Figure 3F**).

**Figure 4:**
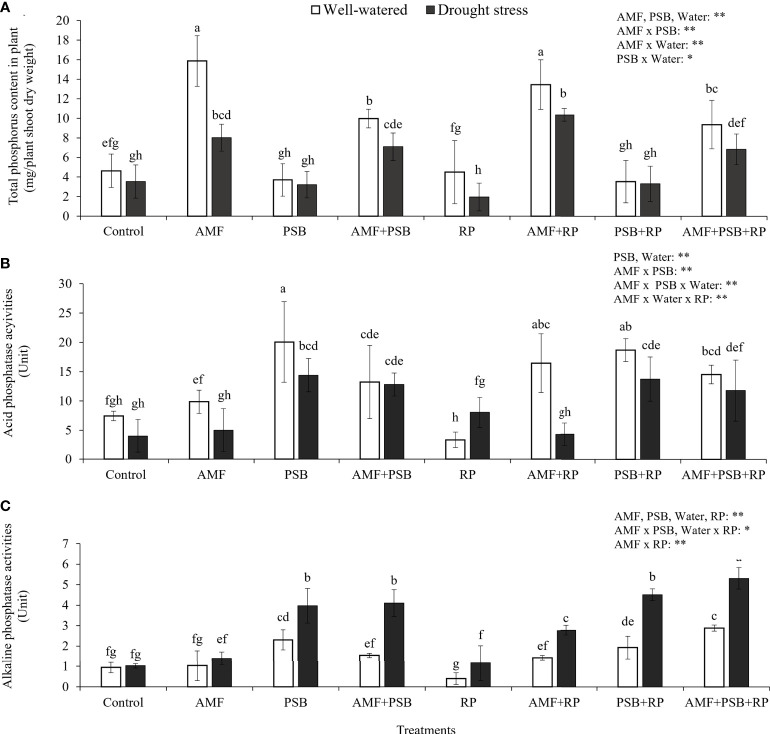
Total phosphorus uptake by plants **(A)**, acid phosphatase **(B)** and alkaline phosphatase enzyme activities **(C)** in rhizosphere soil under well-watered and drought conditions evaluated at 60 DAT. Different letters indicate significant differences at P<0.05 by LSD. Treatment means are the average of four replications. The significant effects of AMF, water, PSB, RP and their interactions are presented as the F value with test of significance (*P< 0.05; **P< 0.01) of four-way ANOVA. Non-significant factors are not shown.

Plant P content was increased (+185%) after addition of AMF and reduced (-25%) after addition of PSB. However, addition of PSB to the treatment without AMF inoculation did not have an effect on plant P content, whereas addition of PSB to the mycorrhizal treatment reduced plant P content significantly (-30%), causing a significant AMF × PSB interaction and hence negative synergy. Plant P content was also higher with higher water availability (**Figure 4A**). The production of phosphatases was especially increased after addition of PSB and was higher in DS than in WW (**Figures 4B, C**). The level of acid phosphatase activity, varying between ~5-20 U, was significantly higher than alkaline phosphatase activity (~1-5 U), a result consistent with the *in vitro* experiment (**Figure 1**).

Correlations between fractional mycorrhizal root colonization, PSB population and soil moisture content with plant growth parameters and production of sunchoke are presented in **Supplementary Table 2**. AMF colonization rate had a negative correlation with soil moisture, MDA and EL of plants, while it had a positive correlation with SPAD value, total chlorophyll concentration, leaf area, biomass, total soluble sugar, tuber fresh weight, tuber dry weight and root volume. The PSB population in soil had a positive correlation with SPAD value, acid and alkaline phosphatase activities and inulin concentration. Soil moisture content had a negative correlation with SPAD value, total chlorophyll concentration, leaf area, biomass, total soluble sugar, alkaline phosphatase, tuber fresh weight and tuber dry weight. In contrast, soil moisture had a positive correlation with %EL of sunchoke. All results suggest that AMF was the dominant factor affecting plant growth performance and yield compared to PSB.

## 4 Discussion

### 4.1 Characterization of phosphate solubilization and the production of plant growth promoting substances of PSB under osmotic stress

Phosphate solubilization capability of PSB has frequently been assessed using the halo zone-based technique. However, this technique is not always reliable due to variation in diffusion rates of different organic acids (Behera et al., 2017b). We therefore complemented the test by actually measuring the available P concentration in PKV broth. Because our intention was to employ PSB to stimulate P solubilization, we evaluated P availability at various levels of osmotic stress. Based on performance with addition of 20% or 30% of PEG-6000, we selected strain KKUT8-1, which was identified as *B. vietnamiensis*, for subsequent experimenting. HPLC data showed that this strain produced various organic acids including gluconic, tartaric, D-malic, L-malic, malonic, lactic, maleic, citric, and succinic acid. Furthermore, it also produced ACC deaminase under osmotic stress (20% PEG), which is further evidence for drought tolerance of this strain. Our results agree with Ali et al. (2014) who found that drought-tolerant bacterial isolates were positive for ACC production under drought. Moreover, Zahir et al. (2008) claimed that inoculation with bacteria capable of producing ACC deaminase could be helpful in eliminating the inhibitory effects of drought in plants. This is because ACC deaminase can convert ACC, an immediate precursor for ethylene production during drought, into ammonia and α-ketobutyrate (Ali et al., 2014).

We also observed production of EPS and IAA by strain KKUT8-1 under osmotic stress. The production of EPS could help bacteria survive drought because EPS can enhance water retention and regulate the diffusion of organic carbon sources (Ali et al., 2014). Moreover, EPS can help PSB to attach to and survive in the soil under drought due to the involvement of a fibrillar material network that permanently connects bacterial cells to root surface (Bashan et al., 2004). The ability of strain KKUT8–1 to produce IAA in the absence or presence of drought is in agreement with the report of Wang et al. (2014), who found that *Serratia* sp. 1–9 had the ability to produce IAA in culture medium containing 20% PEG and without PEG. Additionally, a strain of *B. vietnamiensis* produced the phytohormone IAA when grown with L-tryptophan (Xin et al., 2009). Although many strains of *B. vietnamiensis* have been reported to produce phytohormones, solubilize P and resist various stress conditions (Xin et al., 2009; Park et al., 2010), there is still no application of this bacterial species in enhancing growth and yield of sunchoke until this present study.

Strain KKUT8-1 accumulated proline intracellularly more than released it extracellularly. Moreover, total intracellular proline was higher when cells were grown with PEG than without PEG. This might be because proline is not only produced to maintain cell integrity during growth under stress, but also a common product from amino acid synthesis during cell growth under non-stress (Kishor et al., 2015). Marulanda et al. (2009) reported that *Pseudomonas putida* exhibited high osmotic tolerance and also showed an increase of proline concentration in response to osmotic stress. In addition, accumulation of proline can lower the water potential of the cytoplasm and maintain cell turgor, thus preventing degenerative processes (Kishor et al., 2015).

### 4.2 Interaction between AMF and PSB on promoting growth of sunchoke under well-watered and drought conditions

At the harvest stage, %AMF colonization in plants inoculated with AMF was significantly higher than that in the control plants under both conditions. This result indicates successful colonization of our AMF. Equally, inoculation with PSB resulted in higher bacterial abundance, an effect further enhanced by addition of RP, again indicating successful inoculation. The presence of AMF had significantly positive effect on the growth performance and production of sunchoke. Khaekhum et al. (2021) reported that the use of a single inoculation of AMF *Glomus etunicatum* UDCN52867 g.5 and co-inoculation of endophytic fungi and AMF promoted sunchoke yield production, as evidenced by tuber yield, yield components (number of tubers and tuber size), harvest index (HI), and tuber inulin concentrations compared to plants that were treated with mineral fertilizer.

Whereas inoculation with AMF strongly increased P acquisition and promoted plant performance, the effects of inoculation with PSB were much more limited. Inoculation with PSB increased SPAD values, chlorophyll, and inulin concentration. Positive effects of PSB on SPAD values and chlorophyll concentrations have also been reported by Panhwar et al. (2011) in rice. Inoculation with PSB significantly reduced MDA concentration, an effect similar to that of AMF. Several studies have shown that MDA concentration in mycorrhizal plants is correlated with drought tolerance (Abbaspour et al., 2012; Rani et al., 2018). Rahimzadeh and Pirzad (2017b) showed that under water deficit MDA was significantly reduced in plants inoculated with both AMF and PSB. Furthermore, Boutasknit et al. (2020) showed that AMF reduced the accumulation of MDA, leading to rapid recovery after exposure to drought. A reduction in MDA concentrations in inoculated plants might indicate reduced plant cell membrane damage and alleviated lipid peroxidation in plant cell membranes (Latef and Chaoxing, 2011; Sapre et al., 2018). Fouad et al. (2014) suggested that low level of MDA concentration in mycorrhizal plants could increase plant membrane stability due to mycorrhiza-mediated enhanced P uptake and increased antioxidant production in plants. In order to tolerate drought, plants accumulate TSS to regulate the osmotic potential of cells (Wu et al., 2006). AMF colonization and PSB inoculation enhanced TSS concentration, and a significant interaction was also observed. Our results agree with the study of Subramanian et al. (2006) who found that AMF colonization enhanced TSS concentration of tomato fruits under mild DS and WW, but not under severe drought. Apparently, AMF can maintain a high level of TSS concentration regardless of intensity of drought. Abbaspour et al. (2012) also found that TSS concentration was higher in AMF plants grown under well-watered and drought compared to non-mycorrhizal plants. Inoculation with PSB increased activity of both alkaline phosphatases, whereas inoculation with AMF had only an effect on alkaline phosphatase. Comparable results were found by Kohler et al. (2007), who reported that an increase in acid phosphatase activities in the rhizosphere soils of *Lactuca sativa* depended upon inoculation of AMF and PSB either individually or combined. Likewise, Martyniuk et al. (2019) showed that co-inoculation of PSB and AMF increased alkaline phosphatase activity in the rhizosphere. This is likely because the secretion of phosphatases by PSB and/or AMF was a common mode of facilitating the conversion of insoluble forms of organic P into plant-available forms, which enhanced plant P uptake for growth (Asrar et al., 2012).

Inoculation with PSB had no effect on aboveground and belowground plant performance, and, surprisingly reduced P acquisition when plants were co-inoculated with AMF, indicating an antagonistic response, that is, negative synergy, of both microbial groups. Synergy between AMF and bacteria has often been suggested to occur, based on the idea that both groups have different functions, however tests for synergy have less frequently been employed (Larimer et al., 2010). The authors noted that the effects of AMF and N_2_-fixing microbes (rhizobia, actinobacteria) were additive rather than synergistic. Their study did not involve a formal test of potential synergy between AMF and PSB (or PSF). Possible mechanisms for synergy between AMF and PSB include (1) PSB can grow along AMF hyphae, both on the hyphae entering plant roots and on the hyphae growing in the rhizosphere (Ordoñez et al., 2016), which can help PSB to utilize hyphae as a path to access further areas of the soil containing insoluble phosphorus. (2) PSB solubilize phosphate and release phosphate ions from the added RP or from less-available indigenous P sources, into a form that AMF can take up and transport to the plants. (3) AMF exudates and extensive extraradical hyphae create an environment that can influence growth and metabolisms of soil bacteria and other microbes (Gahan and Schmalenberger, 2014). Several studies did report synergy between both functional microbial groups (Kim et al., 2010; Mäder et al., 2011; Ordoñez et al., 2016; Ghorchiani et al., 2018), although formal tests for synergy, by demonstrating a significant interaction term in the AMF × bacterial symbiont in a full factorial experiment, have in many cases not been executed.

In our experiment we observed a significant AMF × PSB for SPAD (where inoculation with PSB or AMF increased SPAD values, but where the positive effect of PSB only occurred in the absence of AMF inoculation, hence demonstrating a less than additive effect), inulin concentration (where the effect of co-inoculation was also less than additive), MDA concentration (where both symbionts reduced MDA concentration but a less than additive effect in case of co-inoculation), proline concentration (where inoculation with AMF reduced proline concentration, inoculation with PSB increased it, and the combined effect was very weak), chlorophyll concentration (a positive effect of both symbionts but a less than additive effect of co-inoculation), TSS concentration (idem), plant P content (where co-inoculated with both microbial symbionts acquired even less P than plants only inoculated with AMF), alkaline phosphatase (where PSB and AMF increased enzyme activity, but the combined effect was less than additive), and acid phosphatase (idem).

Whereas our study did demonstrate synergy, that is, a significant interaction term, between both microbial guilds in nine cases, all cases implied a less than additive effect or even a negative effect of co-inoculation compared to the most effective microbial symbiont. A similar pattern was noted by Larimer et al. (2010) in their meta-analysis of effects of co-inoculation of AMF and N_2_ fixing microbes. The authors noted that plants that were inoculated with both microbial symbionts tended to underperform and did not meet the expected additive effect. This lack of positive synergy is surprising considering the functionally different roles of PSB, which increase the available P pool, and AMF, which acquire P from that pool but do not increase pool size. Our study was not intended to address that question, but we suggest that the focus on functional differences has to be balanced with a focus on potential competition between both microbial symbionts for carbon provided by the plant or space in or around the root system. A further possible explanation includes the properties of the soil used. Available P in our soil was 8.4 mg kg^-1^ soil and maybe this was adequate for full mycorrhizal functioning without additional benefits of P solubilization. Under such cases P solubilization could increase P availability to levels where negative effects on AMF functioning could be expected. While there was no significant AMF × PSB interaction for AMF root colonization or population size of PSB, we noted significant three-way interactions AMF × PSB × water and AMF × PSB × RP for population size of PSB, suggesting more complex interactions in the rhizosphere. A study by Khaekhum et al. (2021) where sunchoke was either inoculated with AMF and/or with endophytic fungi provided evidence for competition between both fungal guilds, with inoculation by endophytic fungi reducing root colonization by AMF.

Next to the positive effect of RP of enhancing populations of PSB after inoculation, two further instances of a significant interaction PSB × PR were observed. Inulin content did not change after RP addition in non-inoculated soils but increased even further in the treatment of PSB inoculation and RP addition, a clear case of synergy. There was also a significant interaction for MDA concentration, where in the absence of PSB inoculation RP reduced MDA concentration, but in the presence of PSB inoculum RP increased MDA concentration, a clear case of negative synergy.

## 5 Conclusions

Our study revealed that inoculation with AMF enhanced overall growth performance of sunchoke plants, with only limited effects of inoculation of PSB. Co-inoculation of AMF and PSB did only have a small effect further enhancing growth performance, indicating that synergy between microbial consortia is less common that often stated in the literature, echoing the results of an earlier meta-analysis by Larimer et al. (2010). The addition of RP and PSB did also not show synergy in most cases, except inulin concentration, suggesting that beneficial effects of co-inoculation with AMF and PSB are not driven by enhanced P solubilization but are likely due by other, possibly hormonal effects, of PSB on plant root growth. Significant interactions between AMF and water indicate that the importance of AMF is even larger under drought conditions, consistent with earlier studies on the role of AMF in conferring drought tolerance. While positive synergy between AMF and PSB hardly occurred and many effects of co-inoculation were even somewhat less than additive, the question remains how important synergy is for the functioning of agroecosystems. When effects of co-inoculation are larger than the effects of either symbiont when inoculated solely, co-inoculation could be considered a management tool. However, clear cases of negative synergy suggest that agroecosystems need to be well understood before co-inoculation can become common practice.

## Data availability statement

The original contributions presented in the study are included in the article/**Supplementary Material**. Further inquiries can be directed to the corresponding author.

## Author contributions

Conceptualization, SN and SB; resources, WS, JE, and SJ; writing—original draft preparation, SN; review and editing, JE, TK, TS, WM, NR, and SB. All authors contributed to the article and approved the submitted version.

## Funding

This research was funded by Thailand research fund (TRF) for financial support through the Senior Research Scholar Project of Prof. Dr. Sanun Jogloy (Project No. RTA6180002). This work was partly supported by the Protein and Proteomics Research Center for Commercial and Industrial Purposes (ProCCI), Faculty of Science, Khon Kaen University.

## Acknowledgments

We thank the Thailand research fund (TRF) for financial support through the Senior Research Scholar Project of Prof. Dr. Sanun Jogloy(Project No. RTA6180002). This research was supported by the Protein and Proteomics Research Center for Commercial and Industrial Purposes (ProCCI), Faculty of Science, Khon Kaen University.

## Conflict of interest

The authors declare that the research was conducted in the absence of any commercial or financial relationships that could be construed as a potential conflict of interest.

## Publisher’s note

All claims expressed in this article are solely those of the authors and do not necessarily represent those of their affiliated organizations, or those of the publisher, the editors and the reviewers. Any product that may be evaluated in this article, or claim that may be made by its manufacturer, is not guaranteed or endorsed by the publisher.
