# Supplementary material for: Promoting growth and production of sunchoke (Helianthus tuberosus) by co-inoculation with phosphate solubilizing bacteria and arbuscular mycorrhizal fungi under drought
Source: Front Plant Sci. 2022 Oct 31;13:1022319. doi: 10.3389/fpls.2022.1022319 (PMC9660246; doi:10.3389/fpls.2022.1022319)
Supplement: Supplementary file 1 [file DataSheet_1.docx]

Supplementary Material

#

# Supplementary Table 1 Phosphate solubilization index (PSI) and concentrations of available phosphorus solubilized by 10 PSB isolates in the presence of 0, 10, 20 and 30% of PEG-6000

| PSB isolates | Phosphate solubilizing  index (PSI) | Available phosphorus (ppm) | | | |
| --- | --- | --- | --- | --- | --- |
|  |  | Concentration of PEG-6000 | | | |
|  |  | 0% | 10% | 20% | 30% |
| KKU-23 | 0.95 | 1310.90 ab | 1190.9 a | 492.67 b | 273.24 b |
| KKU-2.1-1 | 2.25 | 1284.80 abc | 1044.3 bc | 456.47 bc | 0.00 d |
| KKU-8.1 | 2.10 | 1233.40 bcd | 995.5 bc | 492.24 b | 264.93 b |
| KKU-2.2 | 2.10 | 1072.80 e | 1078.9 b | 457.54 bc | 306.42 a |
| KKU-6.2 | 2.00 | 1180.00 cdf | 966.5 c | 416.38 cd | 217.71 c |
| KKU-8.3 | 2.50 | 746.70 f | 1065.3 b | 472.52 d | 243.58 bc |
| KKUT1-1 | 1.20 | 697.00 f | 1030.7 bc | 378.02 d | 257.98 b |
| KKUT3-1 | 1.60 | 1187.30 cd | 961.9 c | 266.81 e | 270.23 b |
| KKUT8-2 | 1.60 | 1368.60 a | 831.4 d | 0.00 f | 0.00 d |
| KKUT8-1 | 1.60 | 1131.70 de | 966.5 c | 631.14 a | 316.93 a |
| % CV |  | 6.89 | 6.09 | 7.61 | 9.62 |
| F-test |  | ** | ** | ** | ** |

# **, Significant difference at P < 0.01; *, Significant difference at P < 0.05; ns, non-significant difference. Data are the means of three replications. Values with similar lowercase letters in each column are not significantly different according to LSD at P < 0.05.

# Supplementary Table 2 Correlation between AMF root colonization, PSB population, soil moisture and plant growth performance evaluated at 60 DAT.

| Correlation | AMF colonization | PSB population | Soil moisture |
| --- | --- | --- | --- |
| Soil moisture | -0.650** | 0.085 ns |  |
| SPAD value | 0.662** | 0.355** | -0.474** |
| Photosynthesis rate (Pn) | -0.023 ns | 0.135 ns | 0.212 ns |
| Water use efficiency (WUE) | 0.227 ns | 0.177 ns | -0.164 ns |
| Proline concentration | -0.282* | 0.262* | 0.097 ns |
| Relative water content | 0.175 ns | 0.272* | 0.145 ns |
| Total chlorophyll concentration | 0.474** | 0.226 ns | -0.402** |
| Leaf area | 0.827** | -0.039 ns | -0.496** |
| Plant biomass | 0.838** | -0.098 ns | -0.564** |
| Total soluble sugar | 0.608** | 0.222 ns | -0.452** |
| MDA concentration | -0.343** | -0.240 ns | 0.244 ns |
| Acid phosphatase | -0.033 ns | 0.561** | 0.134 ns |
| Alkaline phosphatase | 0.211 ns | 0.715** | -0.352** |
| Electrolyte leakage (EL) | -0.732** | -0.269* | 0.443** |
| Tuber fresh weight (TFW) | 0.563** | -0.068 ns | -0.326** |
| Tuber dry weight (TDW) | 0.608** | -0.031 ns | -0.408** |
| Inulin concentration | 0.159 ns | 0.482** | -0.1079 ns |
| Root volume | 0.396** | 0.0184 ns | -0.018 ns |

# **, Significant difference at P < 0.01; *, Significant difference at P < 0.05; ns, non-significant difference
